# RAD52 as a Potential Target for Synthetic Lethality-Based Anticancer Therapies

**DOI:** 10.3390/cancers11101561

**Published:** 2019-10-14

**Authors:** Monika Toma, Katherine Sullivan-Reed, Tomasz Śliwiński, Tomasz Skorski

**Affiliations:** 1 Sol Sherry Thrombosis Research Center and Fels Institute for Cancer Research and Molecular Biology Lewis Katz School of Medicine, Temple University, Philadelphia, PA 19140, USA; monikatoma3@gmail.com (M.T.); katherine.s.reed@temple.edu (K.S.-R.); 2 Laboratory of Medical Genetics Faculty of Biology and Environmental Protection, University of Lodz, 90-236 Lodz, Poland; tomasz.sliwinski@biol.uni.lodz.pl

**Keywords:** synthetic lethality, dual synthetic lethality, RAD52, PARP1, DNA repair

## Abstract

Alterations in DNA repair systems play a key role in the induction and progression of cancer. Tumor-specific defects in DNA repair mechanisms and activation of alternative repair routes create the opportunity to employ a phenomenon called “synthetic lethality” to eliminate cancer cells. Targeting the backup pathways may amplify endogenous and drug-induced DNA damage and lead to specific eradication of cancer cells. So far, the synthetic lethal interaction between BRCA1/2 and PARP1 has been successfully applied as an anticancer treatment. Although PARP1 constitutes a promising target in the treatment of tumors harboring deficiencies in BRCA1/2—mediated homologous recombination (HR), some tumor cells survive, resulting in disease relapse. It has been suggested that alternative RAD52-mediated HR can protect BRCA1/2-deficient cells from the accumulation of DNA damage and the synthetic lethal effect of PARPi. Thus, simultaneous inhibition of RAD52 and PARP1 might result in a robust dual synthetic lethality, effectively eradicating BRCA1/2-deficient tumor cells. In this review, we will discuss the role of RAD52 and its potential application in synthetic lethality-based anticancer therapies.

## 1. Introduction

Over the past years, physicians and scientists have been implementing major changes in medical practice, leading to a shift from traditional “trial and error” approach to personalized therapy based on the individual features of each patient. Targeted anticancer therapy is a promising tool of current medicine, which allows us to improve the effectiveness of the treatment and increase the overall survival of patients diagnosed with cancer. Multiple studies and clinical trials conducted nowadays utilize personalized anticancer therapy as an approach to provide a great chance to fulfill the need for rationally designed selective treatment. Carcinogenesis is a complex process involving the development of genetic instability, which is responsible for the accumulation of mutations and tumor progression [1]. Therefore, a personalized approach would aim to identify these specific changes in the DNA of an individual patient’s cancer cells, which would allow us to specifically target them, and not harm normal cells and tissues.

Maintenance of genome integrity is critical for cell survival. For that reason, double-strand breaks (DSBs), which disrupt DNA continuity, are among the most toxic lesions, often causing mutations and chromosomal aberrations resulting in neoplastic transformation. In human cells, repair of DSBs is carried out by two major mechanisms—homologous recombination (HR) and non-homologous end-joining (NHEJ) [2]. NHEJ is the main repair pathway in quiescent cells, whereas HR works primarily by utilizing the short proximity of the homologous sequence in proliferating cells.

**Figure 1:**
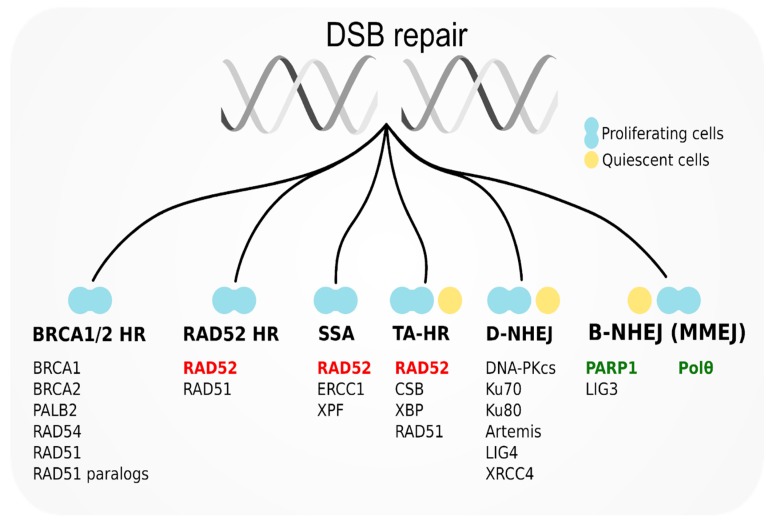
Double-strand break (DSB) repair mechanisms in quiescent and proliferating cells and major proteins participating in them. RAD52 as a potential target for synthetic lethality-based therapy has been marked in red. poly(ADP-ribose) polymerase 1 inhibitors (PARP1) and Polθ—promising partners for dual synthetic lethality have been marked in green.

Canonical DNA-PK-mediated NHEJ (D-NHEJ) is a predominant repair system in normal quiescent cells, while PARP1-mediated alternative NHEJ (B-NHEJ) serves as backup. In normal proliferating cells, PARP1-mediated base excision repair (BER) is employed to prevent replication fork collapse and eventual DSB formation. BRCA1/2-RAD51-dependent HR (BRCA1/2 HR) serves as a main DSB repair pathway and RAD52-RAD51 HR (RAD52 HR) serves as an alternative pathway (Figure 1) [3].

Tumor-specific alterations in DSB repair mechanisms are currently under broad investigation as a novel target for personalized anticancer therapy. Loss of one of the repair pathways is highly probable in cancer cells due to their genomic instability. Under such conditions, cell survival depends fully on the alternative pathway, which compensates for the deficit. Inactivation of the alternative pathway causes synthetic lethality, where the simultaneous loss of function of two genes/pathways results in cell death; however, the individual inactivation of either of these genes/pathways does not affect cell viability [4]. In the context of anticancer treatment, targeting the alternative pathway in tumors that are deficient in the primary repair system will lead to an accumulation of toxic lesions and specific eradication of cancer cells, with low risk for normal cells. Synthetic lethality was first mentioned as a potential tool for anticancer therapy in 1997 by a Nobel Prize winner, Leland H. Hartwell, who understood the great therapeutic potential of this approach as well as the possibilities of using synthetic lethality to identify interaction networks underlying a malignant phenotype [5]. Almost 20 years later, the poly (ADP-ribose) polymerase 1 (PARP1) inhibitor (PARPi) olaparib became the first synthetic lethality-based treatment approved by the Food and Drug Administration (FDA) as a single-agent therapy for BRCA-deficient ovarian and breast cancer patients [6]. While normal cells treated with PARPi are able to efficiently repair DSBs using BRCA1/2-mediated HR, cancer cells carrying defects in HR accumulate high levels of toxic lesions that leads to synthetic lethality and apoptosis [7]. Recently, three other PARPi: rucaparib, niraparib, and talazoparib, obtained FDA approval, and numerous compounds are in various stages of clinical trials as single-agent therapies and in combination with other cytotoxic compounds or radiotherapy [8].

Although currently the synthetic lethal interaction between BRCA1/2 inactivation and PARP1 inhibition is the only one which successfully found application in medicine, the sensitivity of tumors carrying other DNA damage response (DDR) defects to PARPi and to novel inhibitors against new targets for synthetic lethality-based therapy are under investigation [9]. One of the reasons for searching for novel synthetic lethal avenues is that the effect of PARPi is usually short-lived. In many cases, tumor cells become resistant to treatment due to a variety of mechanisms, including secondary mutations restoring function of BRCA1/2, or overexpression of the P-glycoprotein efflux pump, which limits the delivery of the drug into the cells [10]. Additionally, loss of function mutations in 53BP1 or E2F7 may lead to partial restoration of HR and subsequently, the emergence of PARPi resistance [11,12]. Therefore, there is a need to develop a strategy, which would allow for an increase in the effectiveness of PARPi, and the more rapid and robust eradication of tumor cells to eliminate the emergence of PARPi-resistant clones.

It has been suggested that the alternative RAD52-RAD51-dependent HR pathway remains active in BRCA-deficient cells, protecting them from the synthetic lethal effect of PARPi [13]. Since the role of RAD52 appears to be important in BRCA-deficient cells in particular, targeting RAD52 could constitute an attractive anticancer therapeutic approach [14,15].

## 2. RAD52 Protein: Structure and Functional Modifications

During the IVth International Yeast Genetics Conference in 1970, it was proposed that all genetic loci, which mutations confer to X-ray sensitivity, would be given the name “*rad*” followed by an identification number. *Rad52* was first established four years later in *S. cerevisiae,* where its mutation resulted in the abolishment of all recovery processes after irradiation with X-rays [16].

**Figure 2:**
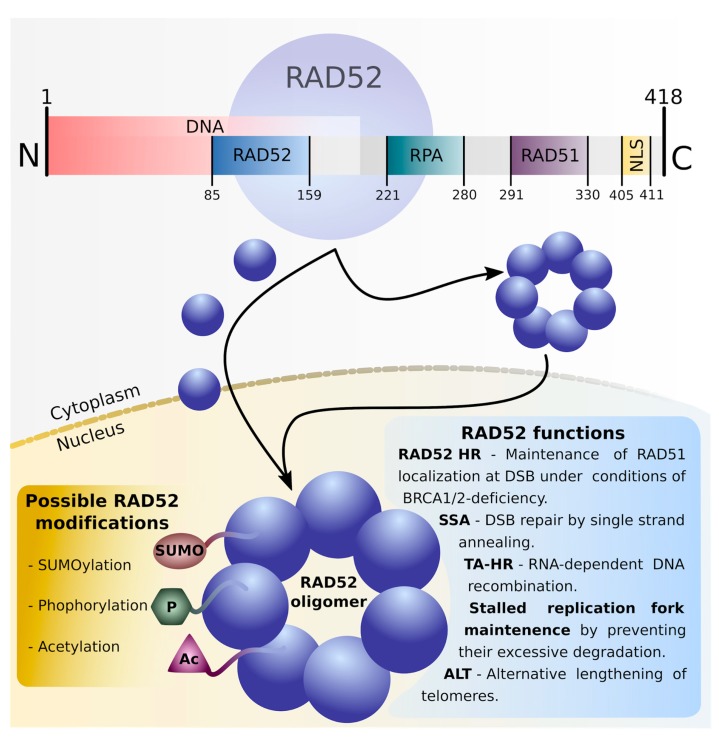
Human RAD52 structure, post-translational modifications, and functions. N-terminal fragment of RAD52 contains a region responsible for its oligomerization and binding with DNA molecule. C-terminal area includes domains interacting with replication protein A (RPA) and RAD51 recombinase, as well as nuclear localization signal (NLS) region responsible for RAD52 transportation to the nucleus. According to the “nuclear retention model”, RAD52 monomer possesses a weak NLS signal allowing only slow transport to the nucleus where RAD52 undergoes oligomerization. The “additive NLS model” suggests formation of RAD52 ring in the cytoplasm, resulting in an additive NLS effect and more robust RAD52 ring transportation to the nucleus. Activity of RAD52 and its participation in different recombination processes can be modulated by post-translational modifications including SUMOylation, phosphorylation, and acetylation.

The crystal structure of purified human RAD52 has suggested it forms a ring-shaped undecamer; however, biophysical studies show that RAD52 in vitro could exist as a heptameric ring with a positively charged ssDNA-binding groove running around the structure (Figure 2) [17,18]. DNA-binding domains have, however, been found on both C- and N-terminal parts of the molecule, therefore supporting speculation of another binding-region outside the first groove of the protein oligomer [18]. RAD52 also contains a residue responsible for its import into the nucleus (nuclear localization signal = NLS) which, in human RAD52, is located at the C-terminal end of the protein [19]. The RAD52 NLS is weak when the protein is in monomeric form and allows for only slow migration into the nucleus; however, in the oligomeric ring structure, the additive effect of seven NLS would allow for more efficient transport to the nucleus. For this reason, the most likely formation of RAD52 heptamer is occurring in the cytoplasm [20]. The N-terminal domain of RAD52 allows for its heptamerization, and it possesses the ability to interact with RAD59. The RAD52/RAD59-dependent recombination pathway appears to be important for the processing of faulty Okazaki fragments [21]. The C-terminal and the central domains of RAD52 facilitate recombination “mediatory function” of the protein.

A variety of post-translational modifications including acetylation, phosphorylation, or (SUMO)ylation modulates the function of numerous proteins. The involvement of human RAD52 in HR repair depends on its acetylation by histone acetyltransferases (HATs) p300/CBP [22]. Unacetylated RAD52 dissociates from DSB along with RAD51 recombinase. The acetylation status of RAD52 is maintained by continuous cooperation between HATs and the histone deacetylases (HDACs) sirtuin2 (SIRT2) and SIRT3 [22]. RAD52 can also undergo (SUMO)ylation which does not influence its protein–protein interactions, although it delays recombination by inhibition of DNA-binding and strand annealing activities [23]. SUMO modification also sustains the activity of yeast Rad52 and protects it from degradation [24]. Phosphorylation of RAD52 by c-ABL1 kinase at tyrosine 104 seems to enhance ssDNA annealing activity and inhibit dsDNA binding abilities of RAD52 [25]. Constitutively active oncogenic BCR-ABL1 kinase facilitates nuclear localization of RAD52 and stimulates SSA repair in leukemia cells [26,27].

## 3. Role of RAD52 in DNA Repair

RAD52 is able to bind ssDNA, facilitating a major role in single strand annealing (SSA) and HR repair of DSBs based on the homologous strand. RAD52 can also operate on single-ended DSBs, preventing excessive degradation of stalled replication fork by converting them into a compact conformation that is less available for reversal enzymes [28]. In checkpoint-deficient cells, RAD52 reverses stalled replication forks to the form in which they can be cleaved by the MUS81/EME1 complex during the process of break-induced replication (BIR) [29]. In fact, RAD52, through its ssDNA annealing activity, is suspected to assemble a displacement loop (D-loop) which invades the homologous chromosome and allows for BIR progression on the template of the homologous sequence [30]. RAD52 can also prevent chromosome end exposure by copying telomere caps from other chromosomes in a subtype of HR—alternative lengthening of telomeres (ALT) [31].

### 3.1. Homologous Recombination (HR)

Although in *S. cerevisae* RAD52 is a predominant recombination protein acting alone in facilitating RAD51 loading onto ssDNA, in mammals its role seems to be diminished by other proteins, namely BRCA1/2. *Rad52-/-* mice are viable, fertile, and show only a slight decrease in HR activity [32]. However, overexpression of RAD52 in mammalian cells enhanced their resistance to ionizing radiation, indicating the importance of RAD52 in the DNA damage response [33]. It has been demonstrated that in the absence of the BRCA1/2-dependent HR pathway, cell viability may be dependent on RAD52-RAD51, indicating that in mammalian cells, HR operates with at least two alternative sub-pathways: BRCA1/2-dependent canonical mechanism and RAD52-dependent alternative repair [34,35,36]. In the latter, RAD52 interacts with RAD51 and places it on RPA-coated ssDNA overhangs, which is possible thanks to the strong inhibitory effect that RAD52 exerts on the RPA-ssDNA complex. It has been suggested that once RAD51 is localized at the DSB, most of RPA and RAD52 are displaced from the DNA; however, some persist surrounded by recombinase filaments, possibly stabilizing further steps of HR [37,38].

### 3.2. Single Strand Annealing (SSA)

In general, DSB end resection and the creation of single-stranded overhangs is a pivotal moment of DNA repair which allows for the cell to choose between not only NHEJ and HR, but also between HR and SSA depending on how far resection has proceeded [39,40]. SSA events require sufficient resection to have direct sequence repeats presented in the form of ssDNA. 53BP1 is a factor that is responsible for the suppression of BRCA1-mediated end resection and the promotion of D-NHEJ. It has been suggested that the absence of 53BP1 leads to hyper-resection of DSBs in G2/S phase. This stage leads to the switch from error-free HR to mutagenic RAD52-mediated SSA. Therefore, cells lacking BRCA1 and 53BP1 require RAD52 for the maintenance of DSBs [41]. In addition, other factors—BRCA1, RNF168, RIF1, histone H2A.X—which inhibit end resection, were shown to suppress SSA [42,43,44].

In SSA, 5’ to 3’ end resection within tandem repeats exposes about 25 nt ssDNA overhangs. RAD52 interacts with RPA-coated overhangs and aligns the complementary regions. It was suggested that after finding initial homology, a further search for stronger interactions and more extensive homology continues without complex dissociation. The alignment occurs due to the overlapping of nucleoproteins present on the opposite sites of DSB [45]. After final homology is achieved, the endonucleolytic complex ERCC1/XPF, in cooperation with RAD52, trims 3’ overhangs. Final gap filling and strand ligation follow this step. SSA often results in the generation of deletions during the step where 3’ ssDNA overhangs are trimmed. Additionally, since SSA uses as templates repetitive elements that are present in multiple other genetic loci, SSA may also lead to translocations [46].

### 3.3. RNA-Dependent DNA Recombination

Although HR is mostly active during G2/S phase due to the short proximity to homologous sequence of sister chromatid or homologous chromosome, it appears that a HR sub-pathway that uses RNA transcripts as a template is active at transcriptionally active regions during G1/G0 phase of the cell cycle. RNA polymerase II can bypass different base modifications, however single strand breaks (SSBs) and DSBs result in permanent blockage of the enzyme. Such damage in transcriptionally active regions is expected to be more toxic than in any other genome area [47].

Under conditions of low abundance of BRCA1/2 during G0 and early G1 phase, its task of RAD51 recruitment to a DSB is fulfilled by RAD52 [48,49,50]. It appears that RAD52 may not only show affinity to ssDNA but also to RNA, and it might be active in repair mechanisms in differentiated, non-dividing cells [49]. Cocaine syndrome B protein (CSB) is expected to be the key protein in transcription-coupled homologous recombination (TC-HR). It detects stalled RNA polymerase and interacts directly with HR proteins RAD51C and RAD52, directing them to DNA damage in coding regions [51]. RAD52 binds to R-loops, which are three stranded DNA-RNA hybrids that allow for repair on the template of RNA transcript. In transcription-associated homologous recombination repair (TA-HR**)** RAD52 is recruited to the RNA-DNA hybrid at the DSB and promotes ERCC excision repair 5 (XPG)-mediated processing, leading to HR-based repair [52].

Two models indicate how RAD52 might promote RNA-mediated repair. In the first, RAD52 directs RNA to the DSB, where it finds homologous sequences with both its termini, creating a synapse that conjoins the ends. In the second model, RAD52 creates an RNA-DNA hybrid at 3’ ssDNA overhang. The overhang created by RNA is then used as a template for reverse transcription, before finally being degraded by RNase H. In the final steps, homology between the created ssDNA and the other end of the DSB allows for end joining and RAD52-promoted SSA [53].

It has been established that not only yeast, but also human RAD52 promotes RNA-templated DNA repair. RNA could constitute a stable template for DSB repair in differentiated cells that do not undergo divisions thus do not have sister chromatid as a template [50,51,54].

## 4. Synthetic Lethality Targeting RAD52

*BRCA1* and *BRCA2* are tumor suppressor genes in which mutations have been widely correlated with hereditary and sporadic breast and ovarian cancer [55]. BRCA1 is responsible for directing DSB repair pathway choice towards recombination-based mechanisms and interacting with proteins participating in end resection. The recombination mediator BRCA2 contains ssDNA, dsDNA, and RAD51-binding domains, which facilitate the formation of recombinase RAD51-ssDNA filament during HR repair [56].

In contrast to the severe effects of RAD52 depletion in yeasts, only a mild effect on recombination was observed in RAD52-deficient vertebrate cells, and *Rad52-/-* mice are viable and fertile [32,57]. However, cancer cells that are deficient in BRCA substitute its activity with RAD52, which, thanks to its ssDNA and RAD51-binding sites, is able to manage HR in a BRCA-independent manner [34]. The fact that RAD52 is essential in human cells only under conditions of BRCA-deficiency makes it an attractive target for synthetic lethality-based anticancer therapy. Such approach is based on natural genetic interactions between DNA repair mechanisms and it utilizes cancer-specific defects. Targeting RAD52 in BRCA-depleted cancer cells will sensitize them to the toxic effect of DSBs, while normal cells and tissues with intact BRCA1/2-dependent HR should not be influenced.

It has been reported that one probable mechanism of synthetic lethality in RAD52/BRCA1/2-depleted cells is the activity of the endonuclease/exonuclease/phosphatase family domain containing protein 1 (EEPD1) [58]. In cells depleted in BRCA, 5’ endonuclease EEPD1 can nick stalled replication forks independently of BRCA to initiate end resection by EXO1, creating the ssDNA 3’ overhangs that are required for HR [59]. Downregulation of EEPD1 results in the suppression of synthetic lethality in RAD52/BRCA1/2-deficient cells. EEPD1-mediated cleavage of stressed replication forks creates a toxic intermediate, which under conditions of impaired BRCA- and RAD52-dependent HR, is dependent fully on error-prone systems for repair, resulting in accumulation of lethal damage in the cell’s genome [58]. Additionally, other evidence confirms that the malfunction of factors like BRCA2 or RAD52, which are responsible for the prevention of excessive degradation of stalled replication forks, might also be responsible for the development of toxic intermediates that lead to cell death [28,60,61].

Several laboratories have focused on the development of a small-molecule inhibitor of RAD52 (RAD52i), which could be utilized to trigger synthetic lethality under conditions of depleted BRCA1/2.

### 4.1. F79

In 2013, the first paper was published that confirmed the successful inhibition of RAD52 in human BRCA1/2-deficient leukemia cells derived from patients [14]. The goal was achieved by the utilization of synthetic peptide aptamer F79 that probably interferes with DNA binding by RAD52.

The authors proposed to use the F79 aptamer to treat leukemias displaying low levels of BRCA1/2. To test this hypothesis, they conducted an analysis of the response of different leukemia types displaying a variety of mutations that cause a low level of one of the members of BRCA-HR pathway, which includes: BCR-ABL1-positive chronic myeloid leukemia (CML) cells in which BRCA1 is downregulated [62], PML-RAR-positive acute promyelocytic leukemia (APL) cells with downregulation of RAD51C (RAD51 paralog), and samples from leukemias which express low levels of BRCA1/2 due to unknown mechanisms. Therefore, another goal of the research was to identify a cohort of patients who could potentially benefit from targeting RAD52.

F79 was able to selectively eliminate BRCA-deficient leukemia cells, with low risk for normal cells. It resulted in synthetic lethality in leukemias carrying BCR-ABL1 and PML-RAR oncogenes as well as epigenetic modifications resulting in BRCA-ness. In vivo tests showed significantly extended life spans of F79 treated SCID mice carrying BCR-ABL1—positive leukemia. F79 treatment resulted in synthetic lethality in *BRCA1/2*-mutated breast, pancreatic, and ovarian cancer cells and displayed synergistic effect with approved drugs such as imatinib (approved for BCR-ABL1-positive leukemia) and ATRA (for PML-RAR-positive leukemia) [14].

### 4.2. 6-OH-dopa

Another small-molecule RAD52i is 6-hydroxy-dopa (6-OH-dopa) [63]. 6-OH-dopa disrupts formation of the RAD52 heptameter superstructure and its dissociation, leading to abolished recruitment of RAD52 to DNA damage sites. 6-OH-dopa was reported to specifically inhibit SSA but had little to no effect on HR or D-NHEJ in BRCA-proficient cells. It selectively halted the proliferation of BRCA1-depleted triple negative breast cancer (TNBC) cells. Selective growth blockage after treatment with 6-OH-dopa was also observed in BRCA-deficient AML and CML cells derived from patients. BRCA-deficient cells treated with the inhibitor demonstrated increased level of DNA damage, thus resulting in increased apoptosis [63].

6-OH-dopa is a dopaminergic toxin derivative and has been reported to contribute to Parkinson disease and degeneration of mitral neurons [64]. Therefore, it is unlikely that 6-OH-dopa finds application in anticancer therapy.

### 4.3. A5MP and AICAR/ZMP

Another screen of libraries of drug-like compounds and FDA-approved drugs identified two substances, which were able to inhibit ssDNA binding by human RAD52. One of the leading compounds—adenosine 5’-monophosphate (A5MP) was able to halt the proliferation of BRCA1-deficient HCC1937 breast cancer cells and did not influence cells with restored BRCA1 expression [65]. The second identified compound—5-Aminoimidazole-4-carboxamide ribonucleotide (AICAR) 5’monophosphate = ZMP is known to imitate A5MP, and similarly to A5MP it disrupts ssDNA-RAD52 binding. Although ZMP is not able to penetrate the cell membrane, its membrane-permeable precursor AICAR is spontaneously phosphorylated intracellularly, generating high levels of ZMP within the cell [66]. AICAR treatment was able to disrupt SSA repair and cisplatin-induced formation of RAD52-ssDNA foci in BRCA1-deficient cells. Both A5MP and ZMP localize at the intersection between two molecules of RAD52, interacting with its DNA-binding domain. Similarly to A5MP, AICAR was able to eliminate BRCA1-deficient HCC1937 cells and BRCA2-deficient Capan1 pancreatic adenocarcinoma cells. BRCA1/2-reconstitution resulted in complete abrogation of the sensitivity to the compound. Ectopic expression of BRCA1 resulted in reversion of sensitivity to AICAR in Capan1 and BCR-ABL1 leukemia cells implicating the synthetic lethal interactions between BRCA-deficiency and AICAR [65].

### 4.4. D-I03

Huang et al. conducted a high throughput screen and identified 17 compounds able to almost completely prevent RAD52-mediated D-Loop formation in vitro tests [67]. The compound D-I03 exhibited the strongest inhibitory effect and preferentially suppressed the proliferation of BRCA-deficient cells in all experimental setups, with no effect in BRCA-proficient counterparts. In BCR-ABL1-positive CML cells which express low levels of BRCA1, treatment led to selective growth inhibition in comparison to BRCA1-proficient control cells [67]. D-I03 led to inhibition of RAD52 but not RAD51 foci formation after cisplatin treatment, and significantly reduced level of SSA repair with no influence on HR. It has been reported that D-I03 binds directly to RAD52 and impairs its DNA-annealing activity [67].

### 4.5. ’1’, ‘6’ and NP-004255

Compounds ‘1’ ((-)-Epigallocatechin) and ‘6’ (Epigallocatechin-3-monogallate) are RAD52i identified in Hengel at al. [68]. They interact directly with the ssDNA binding groove running around the RAD52 oligomer, and disrupt DNA wrapping by RAD52. However, unlike previous inhibitors ‘1’ and ‘6’ do not disrupt the RAD52 ring assembling process. NP-004255 (Corilagin) was identified in the screen of natural products library. It is a macrocyclic ester, which similarly to ‘1’and ‘6,’ binds the RAD52 oligomer in the ssDNA interacting groove, abolishing the activity of this region. Importantly, ‘1’ and ‘6’ compounds were able to inhibit RAD52 binding to RPA-coated ssDNA and its ability to anneal ssDNA. Both compounds were also able to decrease RAD52-dependent BIR in hydroxyurea-treated, checkpoint-depleted cells [29,68]. ‘1’ was able to significantly reduce the viability of BRCA2 or MUS81-depleted cells under conditions of replication stress [68].

### 4.6. F779-0434

Compound F779-0434 exhibits high affinity for RAD52 and stably binds to the protein. F779-0434 interacts with RAD52 residue Lys152, which plays a major role in ssDNA binding. The compound disrupts RAD52-ssDNA interactions and selectively eliminated BRCA2-mutated pancreatic adenocarcinoma Capan1 cells at the concentration range 10–40 µM, whereas the BRCA2-proficient BxPC3 cell line viability was not significantly influenced. The compound could be further investigated as a promising tool for targeted therapy of cancer cells with deficiencies in BRCA1/2-based HR-pathway [69].

## 5. Dual Synthetic Lethality as an Aggressive Anticancer Strategy

**Figure 3:**
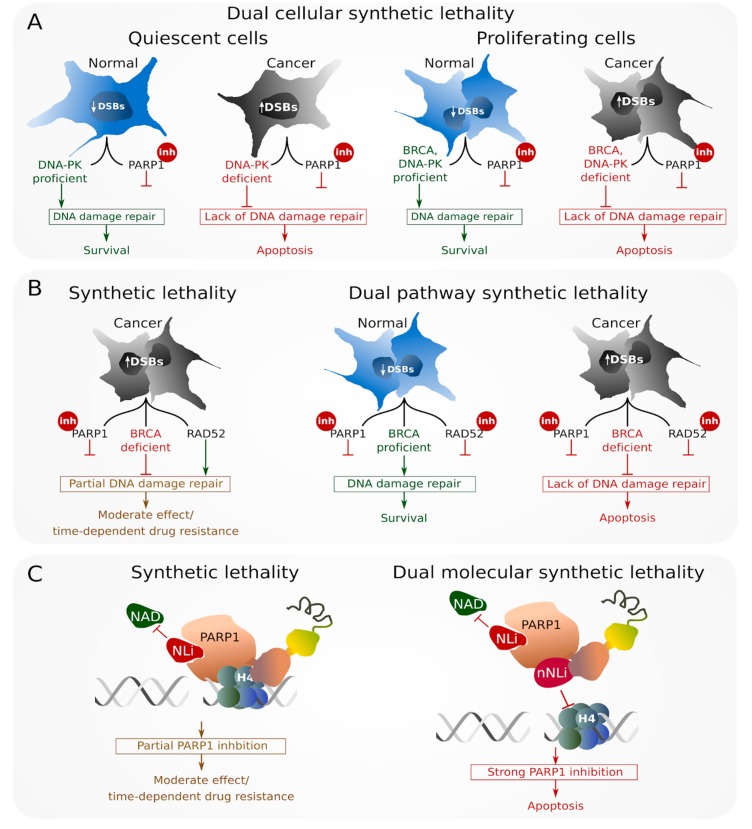
Dual synthetic lethality strategies: (**A**) Dual cellular synthetic lethality—inhibition (inh) of PARP1 gives a chance to simultaneously eliminate DNA-PK (DNA-dependent protein kinase)—deficient quiescent and BRCA (breast cancer susceptibility protein)-deficient proliferating cancer cells accumulating high numbers of DSBs, with no harm for BRCA-proficient normal cells; (**B**) dual pathway synthetic lethality—simultaneous inhibition of RAD52 and PARP1 exerts synergistic synthetic lethality effect against BRCA-deficient cancer cells with no toxicity to BRCA-proficient cells; (**C**) dual molecular synthetic lethality—anti-PARP1 activity of the combination of NAD-like inhibitor (NLi) reducing the catalytic activity of PARP and non-NAD-like inhibitor (nNLi), which abolishes activation of PARP1 by histone H4, resulting in synergistic effect eliminating BRCA-deficient cancer cells.

Most recently, a new strategy named “dual synthetic lethality” emerged from the idea of simultaneous aiming at two targets: (A) Two different types of cancer cells (D-NHEJ—deficient quiescent cells and BRCA1/2 HR—deficient proliferating cells) = “dual cellular synthetic lethality” (Figure 3A) [70], (B) two different repair pathways within the cell (PARP1 and RAD52) = “dual pathways synthetic lethality” (Figure 3B) [71] and (C) two different functions of the same protein (the NAD binding niche of PARP1 and histone 4-mediated activation of PARP1)—“dual molecular synthetic lethality” (Figure 3C) [72]. “Dual synthetic lethality” is an aggressive anticancer approach, which has a chance to increase the effectiveness of cancer cell elimination, thus preventing the emergence of drug-resistant cells [71].

### 5.1. Dual Cellular Synthetic Lethality

Tumor bulk is a heterogeneous agglomeration of cells, containing a small cohort of cancer stem cells (CSCs) which display tumor-initiating properties, and give rise to cancer progenitor cells (CPCs) which constitute the majority of cancer’s mass. CSCs consist of proliferating and quiescent cells. Most of the currently available drugs fail to eradicate CSCs due to their chemo- and radiotherapy-resistance associated with quiescence, and their ability to reproduce tumor mass from even a small number of tumor-initiating cells [73]. Dual cellular synthetic lethality offers the possibility to eradicate quiescent and proliferating CSCs and proliferating CPCs (Figure 3A). PARPi was able to cause extensive “dual cellular synthetic lethality,” simultaneously eliminating DNA-PK-deficient quiescent leukemia CSCs and BRCA1/2-deficient proliferating CSCs and CPCs [70]. Thus, “dual cellular synthetic lethality” is a strategy of great potential allowing for the eradication of therapy-refractory cancer cells utilizing specific vulnerabilities to DNA repair inhibitors.

### 5.2. Dual Pathway Synthetic Lethality

At this time, only PARPi are being applied in personalized anticancer therapy of BRCA-deficient ovarian and breast cancer patients. However, the effect of PARPi is usually temporary and the majority of patients develop therapy resistance. “Dual pathway synthetic lethality” expands the synthetic lethal approach to simultaneous targeting of two repair mechanisms. RAD52 maintains residual HR in BRCA-deficient PARPi-treated cancer cells, so simultaneous targeting PARP1 and RAD52 represents an attractive therapeutic approach [71].

*Rad52-/-Parp1-/-* mice are normal but show delay in the appearance of BRCA1-deficient leukemia when compared to single knockout mice [71]. Simultaneous inhibition of PARP1 and RAD52 by small molecule inhibitors resulted in the synergistic accumulation of lethal DSBs and complete elimination of BRCA1/2-deficient cell lines in comparison to individual agent treatment (Figure 3B). Combination of PARPi and RAD52i also effectively eliminated primary leukemia cells displaying “BRCA1/2-ness”, while individual compounds generated only a partial effect. “Dual pathway synthetic lethality” could be even enhanced by addition standard therapeutic drugs (e.g., imatinib or daunorubicin). Additionally, combination of PARPi and RAD52i inhibitors exerted a synergistic effect against BRCA-deficient tumors in immunodeficient mice with low toxicity to normal cells and tissues [71]. Therefore, “dual pathway synthetic lethality” simultaneously targeting PARP1 and RAD52 offers a promising and very aggressive therapeutic approach against HR-compromised tumors, allowing for more robust elimination of cancer cells and preventing the emergence of drug resistance.

PARPi could potentially be used in combination with inhibitors of other pathways. Polθ (encoded by *PolQ*), is a unique DNA polymerase that contains a helicase-like domain at its N-terminal end. Polθ plays an essential role in B-NHEJ, particularly in microhomology-mediated end-joining (MMEJ) [74]. However, it has been recently suggested that Polθ might also interact with RAD51 and thus may regulate HR repair [75]. The expression of Polθ is relatively low in normal human cells; however, its elevated expression is often associated with poor prognosis in breast cancer [76,77]. Simultaneous inactivation of HR factor *Fancd2* and *Polq* in mice resulted in embryonic lethality, whereas knockdown of Polθ in HR-deficient epithelial ovarian cancer (EOC) and breast cancer cells caused increased cell death, therefore suggesting synthetic lethal interactions between HR and Polθ-mediated repair [75,78]. Polθ depletion resulted in increased sensitivity of HR-deficient cells to PARPi, with no effect on HR-proficient counterparts. *Fancd2-/-Polq-/-* mouse embryonic fibroblasts (MEFs) exhibited high sensitivity even to low doses of PARPi [75]. Therefore, the simultaneous targeting of PARP1 and Polθ might constitute an interesting strategy for treatment of HR-depleted tumors; however, this strategy needs further investigation.

### 5.3. Dual Molecular Synthetic Lethality

“Dual molecular synthetic lethality” simultaneously targets two functions within one molecular target (Figure 3C). Although this approach has not been expanded in the context of RAD52, double inhibition of PARP1 has recently been investigated. All currently clinically utilized PARPi are designed to target the nicotinamide adenine dinucleotide (NAD)-binding site on the PARP1 protein [79,80]. This domain is present not only among the PARP protein family but also in many other enzymes utilizing NAD as a cofactor, which means that NAD-like PARP inhibitors can affect the activity of much broader group of proteins, leading to toxic effects. Recently discovered non-NAD-like PARPi seems to be as effective as NAD-like inhibitors, but less toxic [72,81].

5F02 is a non-NAD-like compound which interferes with PARP1 interactions with histone H4, resulting in the inhibition of PARP1 enzymatic activity [81,82]. 5F02 has been proven effective and selective against breast, prostate, and kidney cancer cells; however, the combination of 5F02 with NAD-like inhibitor generated synergistic anti-tumor effect in comparison to each compound administered individually [81]. Combination of NAD-like and non-NAD-like inhibitors was exceptionally effective in vitro and in vivo against BCR-ABL1-positive BRCA1-deficient CML in chronic phase, at the same time causing little or no toxicity to normal cells and tissues [72]. While NAD-like PARPi usually lead to the accumulation of DSBs, non-NAD-like 5F02 did not seem to induce DSBs. Therefore, the course of action of this inhibitor needs further investigation [72].

## 6. Conclusions

The success of synthetic lethality with PARP1 gave hope for the development of highly personalized therapies that take into consideration the molecular uniqueness of each cancer case. Although PARP1 constitutes a promising target in the treatment of tumors harboring deficiencies in BRCA-mediated HR, some tumor cells acquire therapy-resistance and survive, resulting in disease relapse. Targeting RAD52 represents a next step in synthetic lethality-based anticancer therapy. The fact that its activity is limited exclusively to DNA repair and its absence is only lethal under conditions of HR-deficiency makes RAD52 an attractive target for personalized, highly specific anticancer therapy of HR-compromised tumors. Currently available RAD52 inhibitors prevent the creation of the RAD52-DNA complex, which is crucial in all RAD52-mediated processes. Additionally, the recently developed “dual synthetic lethality” strategy might increase the effectiveness and specificity of cancer cell elimination via targeted approach and prevent the emergence of drug-resistant cells. In the context of this strategy, the combination of RAD52i with PARPi has been reported to exert synergistic lethal effect in HR-deprived cells.

## Abbreviations

**ALT**	**alternative lengthening of telomeres**
APL	acute promyelocytic leukemia
B-NHEJ	backup non-homologous end-joining
BER	base excision repair
BIR	break-induced replication
BRCA1/2 HR	BRCA1/2-mediated homologous recombination
CML	chronic myeloid leukemia
CPC	cancer progenitor cell
D-NHEJ	DNA-PK-mediated non-homologous end-joining
DDR	DNA damage response
D-Loop	displacement loop
DSB	DNA double strand break
EOC	epithelial ovarian cancer
HR	homologous recombination
MMEJ	microhomology-mediated end-joining
NHEJ	non-homologous end-joining
PARP1i	poly(ADP-ribose) polymerase 1 inhibitor
RAD52 HR	RAD52-mediated homologous recombination
RAD52i	RAD52 inhibitor
SSA	single strand annealing
SSB	DNA single strand break
ssDNA	single stranded DNA
TA-HR	transcription-associated homologous recombination
TC-HR	transcription-coupled homologous recombination
TNBC	triple negative breast cancer
